# Enterotoxigenic *Escherichia coli*: intestinal pathogenesis mechanisms and colonization resistance by gut microbiota

**DOI:** 10.1080/19490976.2022.2055943

**Published:** 2022-03-31

**Authors:** Yucheng Zhang, Peng Tan, Ying Zhao, Xi Ma

**Affiliations:** State Key Laboratory of Animal Nutrition, Department of Animal Nutrition and Feed Science, China Agricultural University, Beijing, China

**Keywords:** Enterotoxigenic *Escherichia coli*, gut microbiota, pathogenesis, enterotoxin, colonization resistance

## Abstract

Enterotoxigenic *Escherichia coli* (ETEC) is a major cause of diarrhea in children and travelers in developing countries. ETEC is characterized by the ability to produce major virulence factors including colonization factors (CFs) and enterotoxins, that bind to specific receptors on epithelial cells and induce diarrhea. The gut microbiota is a stable and sophisticated ecosystem that performs a range of beneficial functions for the host, including protection against pathogen colonization. Understanding the pathogenic mechanisms of ETEC and the interaction between the gut microbiota and ETEC represents not only a research need but also an opportunity and challenge to develop precautions for ETEC infection. Herein, this review focuses on recent discoveries about ETEC etiology, pathogenesis and clinical manifestation, and discusses the colonization resistances mediated by gut microbiota, as well as preventative strategies against ETEC with an aim to provide novel insights that can reduce the adverse effect on human health.

## Introduction

**Figure 1.**
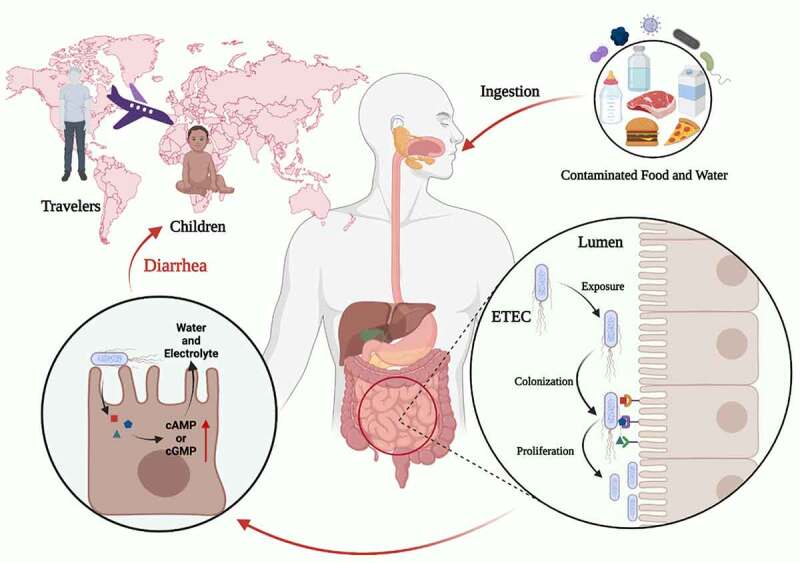
Characteristics of the ETEC infection. ETEC is the major enteric pathogen that account for the diarrhea that occurs in travelers and children in developing countries. ETEC infection is caused by ingestion of contaminated food and water, ETEC through the gastrointestinal tract, and eventually colonization in the small intestine. When ETEC is exposed in the small intestine, it colonizes intestinal epithelial cells via CFs, and ETEC proliferates on the intestinal epithelial after colonization. ETEC produces and delivers heat-labile (LT) and/or heat-stable (ST) enterotoxins to exert toxic effect. Image created with BioRender.

Enterotoxigenic *Escherichia coli* (ETEC) is the major enteric pathogen that account for the tens of millions of diarrheal disease each year.^1^ Children under 5 years are susceptible to ETEC, particularly in endemic areas, which was responsible for an estimated 100 million diarrhea episodes and 60,000 deaths in 2015.^1,2^ ETEC is also the key etiology for traveler’s diarrhea that affects travelers visiting low-income regions of the world, and approximately one-third of all traveler’s diarrhea patients seeking medical care were diagnosed with gastrointestinal disturbance.^3^ ETEC infection is caused by ingestion of contaminated food and water in developing countries, where lack the infrastructure to supply clean drinking water and disposal of excrement. Previous study has shown that ETEC can survive in feces for more than half a year, and generally occur in water in the form of biofilms which provides a greater potential to survive.^4^ (Figure 1) In low-income regions, infrastructure and sanitation associated to people’s health are difficult to dramatically improve in a short period of time, the risk of diarrhea caused by ETEC is hard to be effectively controlled.

ETEC is characterized by the ability to produce heat-labile (LT) and/or heat-stable (ST) enterotoxins.^5^ LT is a high-molecular-weight (84 kDa) enterotoxin with an active alpha subunit surrounded by five identical binding B subunits, ST is a low-molecular-weight peptide consisting of 18 to 19 amino acid residues.^6^ The colonization of ETEC to the surface of the intestinal epithelium is a critical step to exert its toxicity. Apart from the two types of enterotoxins, the colonization factors (CFs) are also major virulence of ETEC.^6^ Once ETEC has colonized in the small intestine epithelia through CFs, effective enterotoxins delivery activity commences, which is responsible for the secretion of water and electrolytes in the intestinal lumen.^7^

In the cases of ETEC infection, the clinical manifestations are characterized by acute watery diarrhea leading to rapid dehydration and prostration within a few hours, which is similar to the clinical manifestations of cholera.^8^ ETEC infection is followed by a variety of symptoms, including vomiting, stomach cramps, headache, and, in rare cases, a slight fever.^9^ Some existing findings suggested that ETEC infection may be associated with some sequelae, such as raising the risk of childhood stunting due to immunological deficiencies and malnutrition, increasing the likelihood of contracting other infectious illnesses, and even influencing cognitive development.^5,10,11^ Furthermore, there is a link between traveler’s diarrhea and post-infectious irritable bowel syndrome.^3^ So far, antibiotics and oral rehydration are the most popular treatments, while antibiotics create a large number of resistant strains and eliminate beneficial bacteria in the gut, making it imperative to find alternative treatments.

ETEC has adapted to its environment through a variety of ways, including a cluster of varied strains that express a variety of CFs to cling to the intestinal epithelium and secrete a variety of enterotoxins. To avoid ETEC infection, it is necessary to thoroughly understand the pathogenic mechanism of ETEC infection and to identify certain targets for developing novel precautions. Although the gut microbiota of mammals performs a variety of beneficial functions for the host, the connection between the gut microbiota and ETEC infection is not well known. This paper reviews the characteristics of ETEC infection in terms of the pathogenic mechanism of major virulence factors, from toxin secretion to eventual diarrhea, as well as the process through which gut microbiota resist ETEC colonization. It also summarizes the available preventive strategies for ETEC infection as reported in recent studies. Our objective is to raise awareness about ETEC infection and to give a more comprehensive view of ETEC pathogenesis that will lead to new strategy for preventing ETEC infection.

## Pathogenesis of virulence factors

Pathogens must adapt to the hostile environment of the gastrointestinal system in order to get nutrients and express virulence factors. In a nutshell, the classical paradigm for ETEC pathogenesis requires two processes to initiate an infection (Figure 1). The first process is ETEC colonizes on the small intestinal epithelium by adhesins, which is necessary for ETEC to release enterotoxin. Secondly, various toxins mainly contain LT and ST enterotoxin, as the essential weapon, released into host epithelia. It is well accepted that both LT and ST produce host diarrhea by increasing cyclic nucleotide synthesis, resulting in electrolyte and water loss (Figure 1).

### Colonization factors

Attachment of ETEC to the small intestinal epithelium is a complex process that is dependent on a variety of CFs located on the bacterial surface. The majority of CFs are fimbria or fimbria-related extracellular filamentous protein polymers known as pili or fimbriae, and the morphology of CFs is classified as fimbrial, fibrillar, helical and afimbrial.^12^ Over 25 distinct types of CFs have been found and described to far (Table 1).^48^ Although ETEC strains can express one or more CFs that have been discovered, around 20–40% of ETEC isolates from clinical patients still have no detectable CFs. This could be due to the true absence of CFs, currently unknown CFs, a dearth of specialized techniques for detecting unknown CFs, and loss of CF properties on subculture of strains.^6,49,50^ With the in-depth development of research, more and more unknown CFs will be investigated clearly. The naming of CFs in the past was complicated and inconsistent. To simplify and standardize the nomenclature of CFs, ETEC CFs are designated by the abbreviation “CS” (Coli Surface antigen), followed by an Arabic numeral. Most of CFs are encoded on intracellular plasmids of ETEC.^51^ The colonization phase is an important node in the process of potentially intervening ETEC infection, and therapies aiming at preventing ETEC colonization have attracted the interest of a significant number of researchers.

**Table 1.** Assembly form and morphology of identified colonization factors

Colonization factor	Assembly class ^a^	Morphology	MW (kDa)	ETEC strain	Accession number	References
CFA/I	CU	Fimbrial	15.0	H10407	M55661	^13,14^
CS1	CU	Fimbrial	16.5	JEF100	CR942285	^15,16^
CS2	CU	Fimbrial	15.3	C91f	Z47800	^17^
CS3	CU	Fribrillae	15.1	PB176	X16944	^18,19^
CS4	CU	Fimbrial	17.0	WS2560B	AY281092	^20,21^
CS5	CU	Helical	21.0	O115:H40	AJ224079	^22–24^
CS6	CU	Nonfimbrial	14.5/16.0	E8775	U04846	^25^
CS7	CU	Helical	21.5	E29101A	AY009095	^26,27^
CS8	Type-IV	Fimbrial	18.0	260–1	AB049751	^28^
CS10	U	Nonfimbrial	16.0	None	None	^29,30^
CS11	U	Fibrillae	None	None	None	^31^
CS12	CU	Fimbrial	19.0	350C1	AY009096	^32^
CS13	CU	Fibrillae	27.0	ESEI_597	OU964063	^33^
CS14	CU	Fimbrial	15.5/17.0	WS3294A	AY283611	^21,29^
CS15	CU	Nonfimbrial	16.3	None	None	^34^
CS17	CU	Fimbrial	17.5	WS6788A	AY515609	^35^
CS18	CU	Fimbrial	25.0	ARG-2	AF335469	^36,37^
CS19	CU	Fimbrial	16.0	WS0115A	AY288101	^38^
CS20	CU	Fimbrial	20.8	H721A	AF438155	^39,40^
CS21	Type-IV	Fimbrial	22.0	E9034A	EF595770	^41,42^
CS22	CU	Fibrillae	15.7	ARG-3	AF145205	^43^
CS23	CU	Nonfimbrial	None	1766a	JQ434477	^44^
CS26	CU	Fimbrial	None	MH2416	HQ203050	^45,46^
CS30	CU	Fimbrial	None	E873	LT174529	^47^
PCFO71	CU	Fimbrial	None	WS2173A	AY513487	^21^

^a^CU, chaperone-usher; U, unknown.

The common classification methods for CFs are morphology, antigenic type, and mode of assembly.^52^ ETEC CFs are classified into two types based on the process of CF assembly: chaperone-usher (CU) pili and Type IV pili. The CU pathway assembles pili was found in a wide range of Gram-negative bacteria, and the majority of ETEC pili are assembled in this manner.^53^ Two proteins are required for the CU pathway to function properly: one is a periplasmic chaperone protein that promotes pilins folding, inhibits their polymerization in the periplasm, and direct them to the usher; the other is an outer membrane protein named “usher” that convenes and coordinates chaperone-subunit complexes forming into a pilus.^54^ Type IV pili (T4P) contribute to a variety of biological processes, including adhesion between host and bacteria, twitching motility, biofilm formation, phage, DNA uptake, and signal transduction.^55^ Two CFs of ETEC, CS8 (CFA/III) and CS21 (longus), belong to T4P assembled by pilin subunits.^12^ T4P systems are similar to type II secretion systems, which translocate the pilin subunit from the periplasm of Gram-negative bacteria into the extracellular environment to form the pilus filament.^53^

Numerous adhesins were identified on the tip of CFs that detect carbohydrate receptors, leading in optimal colonization of the target region. Typically, the receptors for CFs are tested *in vitro* by combing CFs with glycoproteins or glycosphingolipids isolated from intestinal cells. CFs are considered to be species-specific, which explains why ETEC from animals does not induce human infection. Although CS30 can bind to human and porcine intestinal cells via a binding sulfatide, a putative glycosphingolipid receptor, this does not suggest that ETEC isolates carrying CS30 are capable of infecting both people and pigs.^56^ The Global Enteric Multicenter Study (GEMS) has shown that CFA/I and CS1-CS6 were the most common colonization factors, which was examined among ETEC isolates from children under 5 years with moderate-to-severe diarrhea in developing country.^57^ As for traveler’s diarrhea, the previous study detected ETEC isolates causing traveler’s diarrhea in Spanish travelers abroad and showed that the most common CFs were CS21 (58%), CS6 (27%), and CS3 (23%), and EAST1 (65%) and EatA (48%) were the most common nonclassical virulence factors.^58^

### Heat-labile enterotoxin

#### Structures and main features of LT

**Figure 2.**
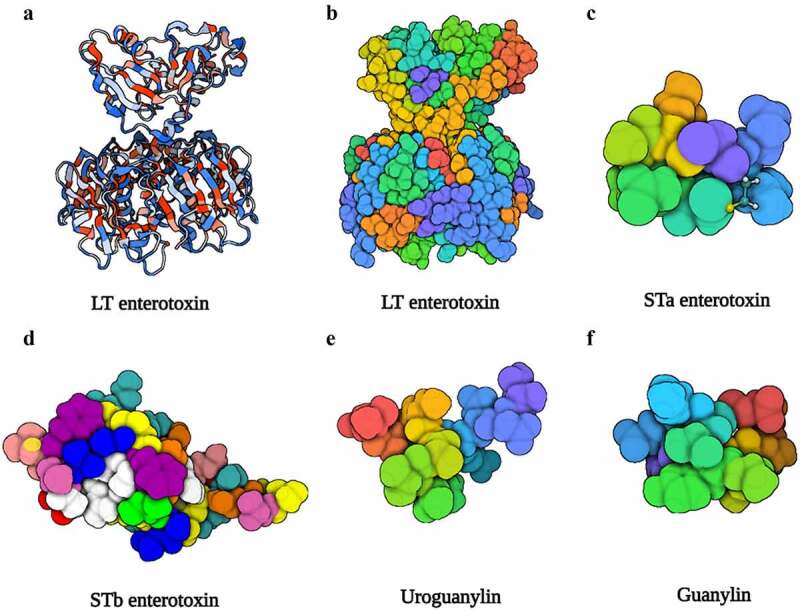
The structure of LT, STa, STb, uroguanylin, and guanylin. (a), (b) Three-dimensional structure of the LT (PDB accession no. 1LTB). (c) Three-dimensional structure of the STa (PDB accession no. 1ETN). (d) Three-dimensional structure of the STb (PDB accession no. 1EHS). (e) Three-dimensional structure of the uroguanylin (PDB accession no. 1UYA). (f) Three-dimensional structure of the guanylin (PDB accession no. 1GNA). Image created with BioRender software.

**Figure 3.**
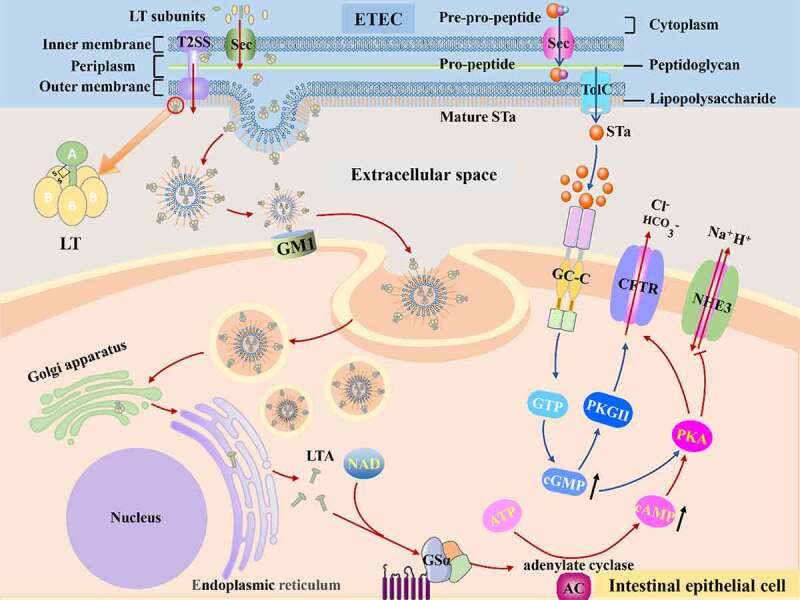
The mechanisms of disease caused by ETEC. Once ETEC established in the small intestinal epithelia via CFs, subsequent efficient enterotoxins delivery activity begins. The ST and LT of ETEC activate adenylyl and guanylate cyclase lead to high level of cAMP and cGMP, which stimulates water and electrolytes secretion in the intestinal lumen.

LT is a high-molecular-weight enterotoxin encoded by the *eltAB* operon on virulence plasmid, and it is structurally and functionally identical to cholera toxin. LT is a heterohexameric molecule composed of a single A subunit that serves as catalytic component and a pentamer B subunit that is responsible for binding to the glycoconjugates on epithelia (Figure 2a).^59^ The A subunit has two domains linked by a disulfide bond (Figure 3): A1 is the active enzymatic activity of LTA and has a stimulatory function on G protein, whereas A2 is regarded as a bridge between the A1 domain and the B subunit, which could anchor the A1 domain into B the subunit.^60^ Additionally, the A2 domain possesses a cell-penetrating function, transporting protein through the membrane to the intracellular regardless of cell types.^61^

According to its antigenic capability and accompanying genetic sequence, LT is categorized into two primary categories (Table 2),^66^ including type I LT isolated from human (LT-Ih) and porcine (LT-Ip), and type II LT (LT-IIa, b, c) predominantly isolated from animals.^67^ Surprisingly, researchers utilized toxicity of LT-II to treat triple negative breast cancer (TNBC) cells, discovering that LT-IIc can cause selective cytotoxicity in TNBC cells but not in non-tumorigenic breast epithelial cells through modulating autophagy.^68^ This finding demonstrates that LT has distinct binding characteristics (Table 2). GM1 ganglioside is the primary receptor of LT-I, and LT-I can bind less stably to GD1b, GM2 and asialo-GM1. The three subtypes of LT-II have separate preferent receptors (Table 2), and LT-II B subunit could bind to toll-like receptor.^62,63,67^ Several residues of LTB directly impact the binding affinity and specificity of LT.^69^ Except for glycolipids on host cells, LT possesses the affinity with lipopolysaccharide (LPS), the main component of cell walls of Gram-negative bacteria.^70^

**Table 2.** Characteristic of LT produced by ETEC

	LTA (amino acid)	LTB (amino acid)	Encoding Gene	Receptors	Host	References
LT-Ih	240	103	*eltAB*	GM1, GM2, GD1b, LPS, asialo-GM1	Humans	^62,63^
LT-Ip	240	103	*eltAB*	GM1, GM2, GD1b, LPS	Pigs	^62,63^
LT-IIa	241	100	*eltAB*	GM1, GM2, GM3, GD1a, GD1b, GD2, GT1b	Buffalo	^62,64^
LT-IIb	243	99	*eltAB*	GM2, GM3, GD1a, GD1α, GM1b, GT1b	Unknown	^62,64^
LT-IIc	241	98	*eltAB*	GM1, GM2, GM3, GD1a, GD1α, GQ1b	Claves	^60,65^

#### Molecular mechanism of LT

Before LT cause toxicity in the host, it must be released from ETEC; protein secretion is required for the procaryotic organism to transfer toxin from the cytoplasm to the extracellular or host cell cytoplasm, which is not a simple task due to the presence of two phospholipid membranes.^71^ ETEC transfer enterotoxin via a Sec-dependent protein secretion system (Figure 3). Above all, enterotoxins rely on Sec secretion systems to be transported across the inner membrane into periplasm, and then enterotoxins are incised and folded into AB5 structure by identifying the N terminal Sec-type cleavable signal sequence, the folded enterotoxin can be transported from periplasm to extracellular milieu in virtue of GspD of T2SS, a large double-β-barrel pore on the outer membrane.^72,73^ T2SS possesses features that span inner and outer membranes and export folded proteins.^74^ Following export, LT bind to lipopolysaccharide (LPS) on the surface of ETEC through the pentamer B subunit.^75^

ETEC has been shown to produce natural Outer membrane vesicles (OMVs) that contain LT and bind to their external surface, and the construction materials of OMVs are similar to outer membrane components.^76^ After OMVs budded, LT located on the external surface of vesicles binds to monosialoganglioside GM1 on the host cells via LTB, forming a tether between host and vesicle.^77^ Thus, LT is primarily delivered by OMVs secretion, and this delivery pathway facilitates LT interaction with host cells. There are several factors that influence LT delivery. EatA, a highly immunogenic secreted serine protease, could degrade adhesin EtpA and then accelerate the delivery of LT.^78^ Another study proved that EatA facilitates the entry of toxins to their receptors by degrading major mucin, which restricts bacteria contact to host cells to a certain extent.^79^ Additionally, previous study demonstrated that highly conserved metalloprotease YghJ, a secreted ETEC antigen, shown the potential to accelerate the efficient delivery of LT by degrading the major mucins in the small intestine.^80^

Subsequently, vesicles were internalized depending on lipid rafts.^81^ Once within the cells, these vesicles transferred LT to the endoplasmic reticulum and cytoplasm. Then, A1 subunit was hydrolyzed by protease and released from A2 subunit. Consequently, A subunit with ADP ribosylating transferase activity catalyzes stimulates G protein α subunit (GSα), activating adenylate cyclase (AC) and leading to the increase of cyclic adenosine monophosphate (cAMP).^82^ Following, cAMP-dependent protein kinase A (PKA) is activated, leading the cystic fibrosis transmembrane conductance regulator (CFTR) being opened. As a result, electrolytes and water are secreted into the intestinal lumen.

#### Other new functions of LT

Additional possible roles for LT are being revealed as study progresses. LT is not only a toxin but also plays a role in facilitating bacterial attachment and intestinal colonization, delivering foreign molecules into cells, and upregulating vaccine antigenicity as an adjuvant.^83^ Additionally, LT was found to have a function in reducing intestinal epithelial viability and inducing intestinal epithelial apoptosis in a time-dependent and dose-dependent manner.^84^ A recent study explored the effect of AB5 toxins including LT on intestinal bacteria and discovered that LTB inhibited the growth of intestinal bacteria capable of mimicking GM1 gangliosides, despite these bacteria may affect human health.^85^ Additionally, research has revealed that LT can inhibit intestinal absorption of vitamin C,^86^ making intestine more vulnerable to be infected.

### Heat-stable enterotoxin

#### Structures and main features of ST

ST-ETEC is the primary cause of infantile diarrhea in underdeveloped nations.^87^ ST is small, non-immunogenic peptide that is opposite to the LT, and classified into two categories based on its structure and function, known as STa and STb (Table 3). STa contains six cysteine residues to form three disulfide bonds and shares a highly sequence and structural similarity with guanylin and uroguanylin, both of them contained two disulfide bonds (Figure 2). STa contains two subtypes consisting of STaH (19aa) which is only isolated from human ETEC strains and STaP (18aa) widely found in porcine, bovine, as well as humans.^88^ STb consists of 48 amino acid peptides and it presents in cattle but not in humans.^90^

**Table 3.** Characteristic of ST produced by ETEC

Enterotoxins	Variants	Amino acid	Encoding Gene	Receptors	Host	References
Heat-stable enterotoxin (STa)	STaH	19	*EstA2, estA3/4, estA7*	GC-C	Humans	^88,89^
	STaP	18	*estA1, estA5, estA6*	GC-C	Pigs	^88,89^
Heat-stable enterotoxin (STb)	STb	48	*estB*	GC-C	Post-weaning piglets	^90,91^

#### Molecular mechanism of STa

The synthesis of STa experiences a complex deformation process that begins with a 72 amino acids pre-pro-peptide precursor generated in the cytoplasm and progresses to a pro-peptide consisting of a signal peptide, a pro sequence, and a carboxy-terminal region, and eventually to a mature peptide (Figure 3).^92^ As a leader peptide, The 19 amino acids signal peptide is proteolytically cleaved first during the transfer process across the inner membrane into periplasm via Sec general export pathway.^93^ Subsequently, the pro sequence is cleaved in the periplasm.^93^ While there is considerable disagreement about this process, one study pointed out that the pro-peptide form can penetrate the outer membrane,^94^ and current research may suggest that some pro-peptide processing occurs outside of bacteria.^95^ Before connecting to their receptors, they must be folded into specific forms analogous to guanylin and uroguanylin on host cells. The specific shapes need two key elements, one is toxin’s structure including cysteine residues, and the other is disulfide oxidoreductase DsbA that catalyzes cysteine residues to form disulfide bonds.^96^ Additionally, the folding procedure also occurs in the periplasm.^96^ After that, mature STa were secreted. STa secretion requires the efflux protein TolC on the outer membrane, according to the previous research.^95^ In addition, EtpA not only accelerated LT delivery, but also essential for effective delivery of ST.^95^

STa performs function through the guanylate cyclase C (GC-C) signal transduction pathway. GC-C receptor is a transmembrane protein, and contains an extracellular domain, a transmembrane region, a kinase homology domain, a hinge region, and a catalytic domain.^97^ STa was identified and coupled to its extracellular ligand binding domain by GC-C receptors expressed on the brush border membrane of small intestine epithelia. This binding activates the intracellular catalytic portion of the GC-C receptors that converts GTP to cyclic GMP (cGMP), resulting in dramatically rise of cGMP.^92^ Accumulation of cGMP mediates diarrhea in two ways. One is opening the CFTR channel by directly activating protein kinase G II (PKGII), directly activating PKA, or indirectly activating PKA by inhibiting phosphodiesterase 3 inhibitor, resulting in a large amount of chloride and bicarbonate release into intestinal lumen.^98–100^ The other is to block the brush border Na/H exchanger NHE3 indirectly, reducing sodium reabsorption, and it has been discovered that the intracellular signaling mechanisms of NHE3 inhibition differ between cell types.^101,102^ Finally, salts ions and water accumulated in the intestinal lumen, leading to ultimate diarrhea. Nevertheless, recent research aiming at another angle, intercellular second messenger signaling, pointed that cGMP outside cell may play a role during infection as ST-induced cGMP were principally exported into basolateral part rather than staying within the cells in human jejunal organoid monolayers, which may complicate the mechanism of ETEC.^103^

According to a recent study, the pathogenesis of ST may be associated with the mucosal metal condition as ST can bind to zinc and iron, and the metal-binding ST weakens greatly to induce cGMP, which may beneficial for host detoxification or good for ETEC to reduce luminal metal concentrates.^104^ In an *in vitro* investigation that cultured human jejunum cells under mechanical forces to create a condition close to the real intestine environment, researchers discovered that flow application increased apical and basolateral cGMP secretion but did not alter intracellular cGMP content.^105^ Because GC-C was a tumor suppressor, some researchers used ST-expressing ETEC to fight tumors, and the results revealed that it reduced the incidence of colorectal cancer by recovering repressed GC-C signal during carcinogenesis.^106^ Furthermore, as GC-C activation is a crucial role in the onset of watery diarrhea, GC-C inhibitors that decrease STa-induced cGMP accumulation may be employed in the development ST-elicit diarrhea therapies.^107^

### Non-canonical virulence factors

#### Tia and TibA

Tia and TibA, two outer membrane proteins, appear to be involved in the initial infection mediated by CFs.^12^ Tia is a 25 kD outer membrane protein encoded on a large pathogenicity Island with a lower GC content in ETEC strain H10407.^108^ Elsinghorst et al. discovered that while ETEC strain H10407 could invade cultured small intestine epithelial cells, it is unable to replicate within the cell.^109^ Subsequent research demonstrated that antisera recognizing Tia blocked invasion by *E. coli* expressing Tia,^110^ indicating that Tia had invasive property in addition to its adhesion function. However, existing researchers have not identified invasion as a characteristic of ETEC, this phenomenon of cell invasion observed *in vitro* requires further investigation.

The *tib* locus consists of four genes, which were *tibDBCA*.^111^ *tibA* encodes a glycosylated outer membrane protein of 104 kD, which is a member of the autotransporter family.^112^ *tibC* encodes a 45 kD heptosyltransferase that glycosylates the TibA precursor by the addition of residues.^111,113^ However, the role of *tibB* and *tibC* is uncertain, although it has been suggested that they participate in gene regulation. The glycosylated form of TibA facilitates the binding of ETEC to the particular receptor on intestinal epithelial cells.^112^ Additionally, TibA promotes bacterial aggregation and biofilm formation, which is independent of TibA glycosylation.^114^

#### EtpA

EtpA, a key secreted adhesin, can serve as a bridge between ETEC and the epithelial surface by attaching to the tips of ETEC flagella and interacting with N-acetylgalactosamine (GalNAc) containing glycans expressed on the intestinal mucosa.^115^ EtpA could interact with flagellin which is required for H10407 to adhere optimally *in vitro* and promotes ETEC colonization in a murine model.^116^ Interestingly, LT can induce the synthesis of mucin MUC2 to intensify the attachment mediated by EtpA.^115^ EtpA is a blood group A-specific lectin/hemagglutinin that interacted with blood group A-related glycans expressed on the surface of epithelia, facilitating bacterial adhesion and effective delivery of ETEC enterotoxins, which explains why severe diarrhea caused by ETEC was more prevalent in blood group A individuals.^117^

#### Other non-canonical virulence factors

Previously published research established that the majority of ETEC, even those with unknown CFs, expressed Type I pili that were highly conserved and coordinated with CFs to enhance adhesion.^118^ Another highly conserved adhesin on the chromosome, EaeH, attaches to the surface of intestinal epithelial cells and assists in ETEC colonization, and the *eaeH* gene expression increases significantly when the host comes into contact with the pathogen.^119^ The *eatA* gene encodes EatA, a member of the serine protease autotransporter of Enterobacteriaceae family, which possessed highly immunogenic.^120^ Recent data suggest that EatA may facilitate ETEC access to intestinal epithelial cells by degrading MUC2.^79^ Apart from the pili related to CFs, LT harbors non-pili adhesins that are straightforward outer membrane adhesins.

## Colonization resistance for ETEC mediated by gut microbiota

Although watery diarrhea is the most prominent clinical manifestation of ETEC infection, not all individuals challenged with ETEC suffer diarrhea symptoms, even though ETEC can be found in the feces and intestinal contents of asymptomatic individuals.^121^ In a previous study, investigators assessed changes in gut microbiota during volunteers challenged with ETEC H10407, and identified some biomarkers based on microbial sequencing data that could predict whether an individual will develop diarrhea following ETEC infection with reasonable accuracy.^122^ According to the researchers, these microbial taxa may help prevent ETEC colonization in the gut. However, these findings are hypotheses rather than conclusions, as too many complicating factors were neglected during the investigation. It is worth recognizing that this discovery inspires a new perspective that gut microbiota may interfere with ETEC infection. The gut microbiota could affect the colonization of ETEC in the small intestine, streptomycin pre-treatment prior to ETEC H10407 inoculation to eradicate normal resident bacterial flora in the intestinal tract, which is required for construction of a mouse model of human ETEC infection.^123^ In addition, the environmental factors of the intestine could also influence the ETEC virulence gene expression, recent work examining the transcriptome of stool samples from volunteers challenged with ETEC H10407, this study demonstrated that ETEC virulence gene expression is likely repressed in the low-oxygen lumen and identified the corresponding transcriptional regulator fumarate and nitrate reduction (FNR) regulator.^124^

**Figure 4.**
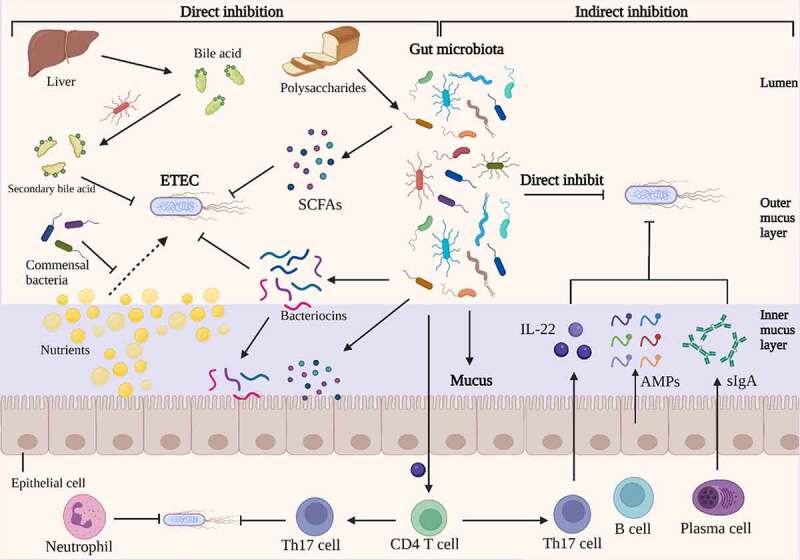
Direct and indirect inhibition mediated by gut microbiota against ETEC infections. On the left, an illustration depicts the direct inhibition against ETEC mediated by gut bacteria. Gut bacteria directly impede ETEC colonization and proliferation. Certain antibacterial compounds, such as bacteriocins, SCFAs, and secondary bile acid, generated by the gut microbiota have been shown to directly inhibit ETEC. Additionally, gut microbiota can compete with ETEC for nutrients, which could limit the growth of ETEC. On the right, indirect methods of competition between gut microbiota and ETEC are depicted. The antimicrobial molecules produced by gut microbiota, such as SCFAs and bacteriocins, which could release into inner mucus layer and stimulate the barrier function. The commensal microbiota induces the differentiation of CD4 T cells into Th17 cells, which contribute to colonization resistance against ETEC by the release of cytokines such as IL-22. Under the stimulation of gut microbiota, intestinal epithelia secrete inflammatory factors, AMPs and sIgA into the mucus, which inhibits the colonization of ETEC. Image created with BioRender.

The gut microbiota is a dynamic and diverse ecosystem composed of trillions of microorganisms that performs a variety of activities, including metabolic regulation, nutritional digestion, immune response regulation, and protection against enteric bacteria.^125–127^ The gut microbiota possessed the ability to inhibit enteric pathogen colonization and expansion, a property referred to as colonization resistance.^128^ Existing research is elucidating how the composition of the gut microbiota can offer resistance to enteric pathogens with the development of next-generation sequencing and metabolomics.^129^ In comparison to other prevalent enteric pathogen, such as *Clostridium difficile, Salmonella enterica serovar* Typhimurium, and Enterohemorrhagic *Escherichia coli*, there are a limited studies on colonization resistance of gut microbiota against ETEC. Colonization resistance against ETEC is achieved by the use of numerous mechanisms that remain poorly understood, however there is evidence that both direct pathogen inhibition and indirect pathogen inhibition via host systems may be involved (Figure 4).^130^

### 3.1 Direct inhibition

Numerous bioactive small molecules produced by the gut microbiota can impede the growth of enteric pathogens, including short-chain fatty acids (SCFAs), secondary bile acids, and Bacteriocins.^131^ SCFAs are saturated fatty acids with a chain length ranging from one to six carbon atoms and are the main metabolites produced by bacterial fermentation of non-digestible polysaccharides in the gastrointestinal tract.^132^ Acetate (C2), propionate (C3) and butyrate (C4) are the most abundant SCFAs in the human body.^133^ The concentration of SCFAs is inversely related to gut pH, which can be altered to prevent the growth of pathogenic *E. coli*.^134,135^ Acetate is the most abundant SCFAs in the intestinal lumen, which has a role in inhibiting the expansion of pathogenic *E. coli* by depleting intracellular methionine pools.^136,137^ A previous study demonstrated that addition of SCFAs into the medium could reduce the production of LT from ETEC, which may be due to the disturbance of the biosynthesis of LT.^138^ Additionally, SCFAs are a primary fuel for the colonic epithelium, and directly affect the health of intestinal epithelia by enhancing intestinal barrier function.^139^ Bile acids are synthesized in the liver and secreted into small intestine to aid in digestion of dietary lipids. In the intestine tract, bile acids are modified by the gut microbiota into secondary bile acids with antimicrobial property.^140^ And bile acids inhibited the binding of ETEC heat-labile enterotoxins to GM1 receptor, mitigating the toxicity effect of LT.^141^

Bacteriocins are bacterially produced peptides that are active against other bacteria and against which the producer has a specific immunity mechanism.^142,143^ Probiotics could produce bacteriocins to facilitate its probiotic function in a number of ways.^142^ For example, bacteriocins may function as antimicrobial peptides, directly eradicating pathogens;^144^ they may act as colonizing peptides, helping the colonization of a probiotic in the intestine trat;^145^ or they may serve as signaling peptides, signaling other bacteria or the immune system of the host.^146^ Mircocins produced by Gram-negative bacteria belong to the large class of bacteriocins.^147^ Probiotic *Escherichia coli* Nissle 1917 could utilize microcins to limit the expansion and colonization of pathogenic *E. coli* in infected mice.^148^

### 3.2 Indirect inhibition

Indirectly, gut microbiota also inhibit pathogen colonization infection by increasing host defense mechanisms, such as promoting mucosal barrier function and enhancing innate immune response.^127^ The gut microbiota has the ability to stimulate the epithelium to create antibacterial compounds and mucus, which serves as the first line of defense against pathogen colonization.^149^ The notion that gut bacteria increase epithelial barrier function is mostly supported by indirect evidence. For example, germ-free mice had decreased antimicrobial peptide synthesis and higher intestinal mucus penetrability in the small intestine.^150,151^ Along with physically preventing pathogens from reaching the epithelial surface, the mucus layer protects the epithelium barrier by storing antimicrobial agents such as antimicrobial peptides and secretory IgA generated by intestinal epithelial cells.^152^ Additionally, by improving the function of the epithelial barrier via metabolites produced by the gut microbiota, such as SCFAs, the epithelium is able to block the translocation of enterotoxins produced by pathogenic *E. coli.^153^*

Not only does the gut microbiota improve mucosal barrier function, but it also boosts host immunity to protect against enteric infection, modifying the host’s vulnerability to diarrheal pathogens. The commensal microbiota stimulates CD4 T cell development into Th17 cells, which may contribute to colonization resistance against enteric pathogen via cytokine production such as IL-22.^154^ The gut microbiota is densely harbored by trillions of bacteria belonging to several hundreds of different species, which making it difficult to investigate the specific function of probiotic in the colonization resistance. The current approach to this challenge is to separate probiotics that play a critical role in diarrhea resistance from feces and chyme and to research their unique mechanisms of inhibition of ETEC infection using *in vitro* epithelial cell co-culture and *in vivo* animal model. In a sterile pig model, *Lactobacillus plantarum* can protect against ETEC infection by decreasing IL-1α and IL-8 expression and increasing IL-10 expression in the small intestine.^155^

## Strategies to prevent ETEC infection

### 4.1 Vaccines

Considering protective immunity against ETEC develops after natural and experimental infection, indicating that vaccine-induced ETEC immunity should be feasible. Vaccination is now recognized as the most effective method of preventing ETEC infection, given public hygiene concerns in developing nations cannot be resolved quickly.^156^ Following vaccination, individuals should be protected from at least common strains.^156^ Interdicting ETEC adhesion to the intestinal surface and neutralizing the toxin are the primary goals of vaccine development, and the vaccine for ETEC is often administered orally. Nevertheless, highly variable of CFs and lack of suitable animal model to evaluate vaccine efficacy make it so complex to design a vaccine that there is no licensed vaccine up to now.^157,158^ Sufficient coverage of CFs remains a significant issue in producing an effective vaccination, and a suitable technique that targets those prevalent adhesins is necessary. According to a previous study, approximately 66% of pediatric moderate-to-severe diarrhea cases caused by ETEC expressing only ST or LT could be prevented in developing countries if effective ETEC vaccine candidates based on major CFs such as CFA/I and CS1-CS6 are developed, and the rate would increase to 77% if CS14 is added to CF-based ETEC vaccine candidates.^57^ Data from a systematic review of ETEC epidemiology also demonstrated that CFA/I-expressing strains were common in all regions (17%), and the results were obtained by analyzing the17205 ETEC isolates abstracted from 136 studies.^49^ In recent years, multi-epitope fusion antigen technology has been employed to develop multivalent vaccinations, which may aid in the development of vaccines against common CFs.^159^

Current research on vaccine target ST is primarily focused on modifying it to eliminate its high toxicity, identifying a protein carrier couple to ST to increase its immunogenicity, and minimizing potential immunological cross-reactivity.^160^ Due to the high immunogenicity of LT as a vaccine antigen, LT is frequently employed as an effective adjuvant or carrier protein for multivalent vaccine development.^161^ Along with multivalent vaccine, another major area of vaccine research is the quest for conserved antigens. A recent study found that EtpA and EatA are high conserved virulence molecules, as they both present in about half of 1159 globally ETEC isolates and do not exhibit obvious regional distribution differences.^162^ ETVAX is the most advanced ETEC vaccine candidate at the moment,^163^ since it consists of four inactivated recombinant *E. coli* strains hyper-expressing CFA/I, CS3, CS5, and CS6 adhesins, along with a recombinant subunit protein LCTBA.^164^ LCTBA is a hybrid B subunit of LT and cholera toxin, previous study demonstrated that cholera toxin B subunit was the immunogen in cholera vaccine which induced cross-protective immunity against LT-producing ETEC,^165^ due to homology between LT B subunit and cholera toxin B subunit.^166^ ETVAX was shown safe and immunogenic in adults from Swedish and ETEC endemic regions.^167,168^ Firdausi Qadri et al. reported that ETVA induced a broad protective response in Bangladesh children older than 12 months.^164^ Additionally, researchers found that ETVAX-induced antibodies to cross-react to CS1, CS14, CS17, and CS7 adhesins, which may result in expanded ETEC strain coverage of ETVAX vaccine.^169^ According to the ETVAX clinic experiment, targeting common antigens to the greatest extent possible may be feasible.^164^ Although ETVAX has demonstrated a favorable preventive impact in clinical trials, the true preventive benefit may be compromised if it does not also develop protective immunity against ETEC strains that produce ST. Data from recent studies suggest that ETEC producing ST is easier to cause moderate-to-severe diarrhea in young children compared with the ETEC producing LT.^87,170^ In addition, previous study reported that over two-thirds of ETEC strains isolated from patients express ST alone or together with LT.^171^ Therefore, a broadly protective vaccine should carry multiple the most common CFAs to induce anti-adhesin immunity and toxoid antigens to induce antitoxin immunity against LT and ST.^156,172^ Above all, the process has been well explored for a long period of time, and researchers have been developing vaccines based on the major virulence.

### 4.2 Specific antibody

Extensive study has been conducted on the usage of antibody that can prevent diarrhea caused by comparable toxin antigens. Evidence demonstrated that volunteers challenged with the ETEC strain B7A, 50% protection against moderate and severe diarrhea was seen in individuals who received bovine serum immunoglobulins targeting to the whole ETEC strain BA7.^173^ In a nonhuman primate trial, oral administration of secretory IgA against colonization factor CFA/I significantly decreased the risk of diarrhea caused by ETEC H10407.^174^ Hyperimmune bovine colostrum (HBC) is produced by repeated immunization of pregnant cows, which is high in specific antibodies and immunomodulatory components, has an effective prophylactic function against gastrointestinal illnesses.^175^ In addition, the use of HBC rich in microbe-specific IgG for the prevention and treatment of gastrointestinal infections is a safe precaution, which is unlike antibiotics, they do not disturb the gut microbiota.^176^ An early clinical study conducted by Tacket et al. showed that daily consumption of an ETEC hyperimmune bovine milk concentrate shortly after each meal protected volunteers from an experimental oral challenge with 1.2 × 10^9^ CFU of ETEC H10407.^177^ In a subsequent study by Otto and colleagues, an ETEC HBC delivered prior to each meal reduced the incidence of diarrhea in volunteers orally challenged with ETEC H10407.^178^ In a controlled human infection model (CHIM) study, volunteers received oral bovine colostrum IgG antibodies against CS17 significantly prevent diarrhea caused by ETEC strain LSN03-016011/A expressing CS17.^179^ Maternal natural IgG antibodies evoked by the maternal microbiota can protect new-born mice lacking the ability to make IgG against ETEC infection, regardless of whether the antibodies are transmitted through the placenta or breast milk.^180^ In view of these considerations, oral delivery of antibody may enhance passive immunization and provide a novel immunoprophylaxis technique that is effective against ETEC. At the same time, the future challenges of specific antibody including stability, inexpensive cost, and availability need to be taken into consideration.^181^

### 4.3 Antimicrobial molecule

Antimicrobial peptides (AMPs), which are synthesized by diverse organisms or synthetically, are used to fight bacterial infection due to their broad-spectrum antimicrobial activity.^182,183^ Saliva antimicrobial peptide histatin-5 was shown to inhibit ETEC adhesion and colonization, demonstrating that saliva components combat pathogens introduced through the mouth, which is a component of the innate immune system.^184^ In an ETEC challenged mouse model, the Lasso peptide Microcin J25 improved epithelial barrier function by increasing tight junctions expression in the small intestine, and alleviated gut inflammatory responses.^185^ Application of AMPs has the potential to be a beneficial and protective method for ETEC infection. Furthermore, AMPs benefited the intestinal barrier function, inflammatory response, and gut microbiota when ETEC was challenge.^186,187^

Additionally, natural products exhibited a variety of beneficial functions, including antibacterial, anti-inflammatory, and antioxidant capabilities. Dietary macleaya cordata plays a preventative role with respect to ETEC infection by alleviating ETEC-induced oxidative stress and enhancing immunological function.^188^ Icariin and its phosphorylated derivatives can help reduce inflammation and oxidative stress produced by ETEC K88 by inhibiting the production of the p38 MAPK.^189^ Certain polyphenol extracts have been identified as potential candidates for preventative method because to their ability to block the conjunction of LT and its receptor.^190^

## Conclusion

The available research demonstrates the mechanism of major virulence factors of ETEC. With the deepening of researches in this filed, more and more novel virulence factors and antigens have been discovered. The identification of specific virulence factors involved in the pathogenic process of ETEC and specific antigens on the surface of ETEC will allow the development of novel vaccine. Given the critical role of the gut microbiota in protection against ETEC, targeting the microbiota to develop preventive strategy has attracted the interest of a large number of researchers. Clearly, considerable work remains to be done not only to gain a greater knowledge of the interaction between the gut microbiota and ETEC, but also to determine the most effective strategy to utilize this relationship to prevent or cure ETEC both inside and outside the intestine.

## Abbreviations

ETEC, Enterotoxigenic *Escherichia coli*; LT, heat-labile enterotoxin; ST, heat-stable enterotoxin; CFs, colonization factors; CS, Coli Surface antigen; CU, Cheperone-usher; T4P, Type IV pili; GalNAc, N-acetylgalactosamine; LTA, heat-labile enterotoxin A subunit; LTB, heat-labile enterotoxin B subunit; LT-Ih, type I LT isolated from human; LT-Ip, type I LT isolated from porcine; LT-IIa, b, c, type II heat-labile enterotoxin a, b,c; TNBC, triple negative breast cancer; LPS, lipopolysaccharide; OMVs, outer membrane vesicles; GSα, G protein α subunit; AC, adenylate cyclase; cAMP, cyclic adenosine monophosphate; PKA, protein kinase A; CFTR, cystic fibrosis transmembrane conductance regulator; STaH, type a ST isolated from human; STaP, type a ST isolated from porcine; GC-C, guanylate cyclase C; cAMP, cyclic adenosine monophosphate; cGMP, cyclic guanosine monophosphate; PKG, protein kinase G II; FNR, fumarate and nitrate reduction regulator; HBC, hyperimmune bovine colostrum; Amps, antimicrobial peptides; FNR, fumarate and nitrate reduction.
